# Leopard density and determinants of space use in a farming landscape in South Africa

**DOI:** 10.1038/s41598-024-61013-6

**Published:** 2024-05-08

**Authors:** McKaughan J.E.T., Stephens P.A., Lucas C., Guichard-Kruger N., Guichard-Kruger F., Hill R.A.

**Affiliations:** 1 Department of Anthropology, Durham University, South Road, Durham, DH1 3LE UK; 2 Conservation Ecology Group, Department of Biosciences, Durham University, South Road, Durham, DH1 3LE UK; 3 Primate and Predator Project, Alldays Wildlife and Communities Research Centre, Campfornis Game Farm, Alldays, South Africa; 4 School of Animal, Rural and Environmental Sciences, Nottingham Trent University, Southwell, UK; 5 Department of Biological Sciences, Faculty of Science, Engineering and Agriculture, University of Venda, Private Bag X5050, Thohoyandou, 0950 South Africa

**Keywords:** Habitat use, Population density, Occupancy models, Human-wildlife conflict, Camera trap, *Panthera pardus*, Behavioural ecology, Conservation biology, Ecological modelling, Population dynamics

## Abstract

Protected areas are traditionally the foundation of conservation strategy, but land not formally protected is of particular importance for the conservation of large carnivores because of their typically wide-ranging nature. In South Africa, leopard (*Panthera pardus*) population decreases are thought to be occurring in areas of human development and intense negative interactions, but research is biased towards protected areas, with quantitative information on population sizes and trends in non-protected areas severely lacking. Using Spatially Explicit Capture-Recapture and occupancy techniques including 10 environmental and anthropogenic covariates, we analysed camera trap data from commercial farmland in South Africa where negative human-wildlife interactions are reported to be high. Our findings demonstrate that leopards persist at a moderate density (2.21 /100 km^2^) and exhibit signs of avoidance from areas where lethal control measures are implemented. This suggests leopards have the potential to navigate mixed mosaic landscapes effectively, enhancing their chances of long-term survival and coexistence with humans. Mixed mosaics of agriculture that include crops, game and livestock farming should be encouraged and, providing lethal control is not ubiquitous in the landscape, chains of safer spaces should permit vital landscape connectivity. However, continuing to promote non-lethal mitigation techniques remains vital.

## Introduction

Land outside protected areas is of great conservation interest due to its potential for a more connected approach that prevents protected areas from becoming isolated “megazoos”^1,2^. Anthropogenic habitat conversion has resulted in a 53% reduction in large carnivore historic range^3^, with natural habitat transformation for livestock and crop farming particularly damaging^4–6^. Large carnivores are typically wide-ranging and so areas not formally protected are of particular importance for their conservation^7,8^. However, these environments often carry greater risk for large carnivores due to direct and indirect threats from anthropogenic activities^9^. Where human activity is greater, such as in urban areas and farmland^8,10,11^, these risks are heightened.

Survival of large carnivores in agricultural areas is influenced by farmer tolerance and the ability of these carnivores to utilise human-modified landscapes^12^. Cultivated lands can both attract and deter carnivore species due to their potential for resources and the perceived risks of human contact, respectively^13,14^, with farmers using legal and illegal removal to protect their livelihoods from animals they consider problematic or a threat^15^. Even in protected areas, carnivores have shown signs of behavioural adaption to minimise human contact, with wolves (*Canis lupus*) in Europe found to concentrate their activity in core areas of protected spaces to minimise interactions with humans and reduce the associated risks, even though there is ample suitable habitat and prey available outside the centre of the protected area^16^. Similarly, lions (*Panthera leo*) in Zimbabwe have been shown to avoid areas of higher risk from humans, particularly the edge of protected areas with prevalent bushmeat snaring and where regular trophy hunting occurs, suggesting risk-based behavioural adjustments^10^. Large carnivores have also been seen to adapt their behaviour temporally in response to human risks. Solitary brown bears (*Ursus arctos*) in Scandinavia were seen to increase their movements after dark once the hunting season started, possibly to compensate for reduced daytime activity when hunters were active^17^. Temporal behavioural adjustments have been particularly notable in urban populations, with leopards (*Panthera pardus*) in India navigating highly populated urban landscapes, but only when human activity was at its lowest^18^.

Leopards are one of the widest-ranging large carnivores, but have experienced a global range contraction of over 30% in the last 20 years, with a 48 to 67% historical range decrease in Africa^19^. Their broad diet is central to their adaptability^20^, underpinning their capacity to utilise modified environments, such as pastoral and crop lands^21,22^ and urban areas^18^. Despite their ecological flexibility, they remain threatened, particularly by habitat fragmentation and destruction, as well as through direct mortality resulting from persecution, snaring and over-harvesting for sport and body-parts^23^.

South Africa’s Limpopo Province is thought to have particularly high leopard population abundance, but 95% of suitable leopard habitat in the province is not formally protected^8^. The rise of game species farming has benefitted many wild species by restoring natural bushland, a major factor in suitable leopard habitat. Nevertheless, this land use brings its challenges, with many larger livestock ranching farms split into smaller, fenced-off properties. Use of (often impenetrable) fencing has grown exponentially, inhibiting many ecological processes and most notably movement^15,24^. Restricting natural movement can reduce population connectivity and lead to isolated populations susceptible to demographic and environmental variations, while interrupting gene flow increases the risk of interbreeding and population fragility^24^, particularly for wide-ranging species. This has been seen in other leopard populations in South Africa, and is of particular concern where densities are lower or there are high levels of human-induced offtake^25^. It has also created a new dynamic of conflict, as many farmers’ livelihoods depend on animals that are natural prey species to large carnivores such as leopards^15,26^.

Limited research has investigated how large carnivores respond to human activity in situations where they do not have access to protected areas as a refuge^11^. With its mixed agricultural land use, as well as the level of leopard persecution^27^ due to real or perceived threats to local livelihoods^28,29^, the Alldays area, Limpopo Province, South Africa, can be considered representative of the wider Limpopo Province^29^. We used data from a camera trap survey to estimate leopard density for the area and to explore anthropogenic and ecological factors influencing leopard habitat use. We discuss these findings in relation to recent work on local landowner perceptions collected through interview data by Lucas et al.^27^. Finally, we outline the significance of these findings for leopard conservation and management across this and similar landscapes.

## Results

The survey took place in the wider land around Alldays, Limpopo Province, South Africa, an area dominated by game farming (for breeding, sales and hunting), with some crop farming, livestock farming, and some eco-tourism reserves. The survey area is dissected by the Mogalakwena River, providing a natural water source to many properties, as well as a safer route for wildlife to pass underneath the main road used by regular agricultural and large mining vehicle traffic. Leopard population density was estimated using Spatially Explicit Capture-Recapture (SECR) and site-use using occupancy modelling. A 2km grid over the survey area was created and 58 camera traps were deployed at 29 stations, using one station per grid square. Stations were an average of 2.04km apart (range 1.5–2.7km).

The camera trap survey comprised 2433 trap nights over a 90-day period, with a mean of 83.9 days active (range: 24–90) for the 29 individual cameras. Leopards were captured at 17 of the 29 sites (58.6%), a total of 58 times.

### Density estimation

We identified 13 adult leopards (eight female, five male) and three juveniles. Identified leopards were captured 51 times. The best fitting model accounted for detector-specific learned responses, producing a leopard density estimate of 2.21/100 km^2^ (95% CI 1.10–4.20) (Table 1, all model results in Supplementary Information, Table S1), with detection probability, *g0* = 0.018 (± SE 0.007; 95% CI 0.009–0.04) and scale parameter, σ = 23.99km (± SE 4.73; 95% CI 16.4–35.2). Female leopard density (1.36/100 km^2^, 95% CI 0.64–2.90) was almost twice that of males (0.85/100 km^2^, 95% CI 0.34–2.10) (Table 1). The mean maximum distance moved was 5871m (± SE 1376).

**Table 1:** Total, male and female leopard density estimates from spatially explicit capture-recapture (SECR) analysis, with standard error (SE), 95% lower confidence interval (LCI) and 95% upper confidence interval (UCI).

	Density /100km^2^	SE	LCI	UCI
Total	2.21	0.74	1.10	4.20
Female	1.36	0.55	0.64	2.90
Male	0.85	0.41	0.34	2.10

### Leopard site use

Independent leopard detections were recorded 55 times. Naïve detection probability was 0.107 and naïve site use probability was 0.623.

**Figure 1:**
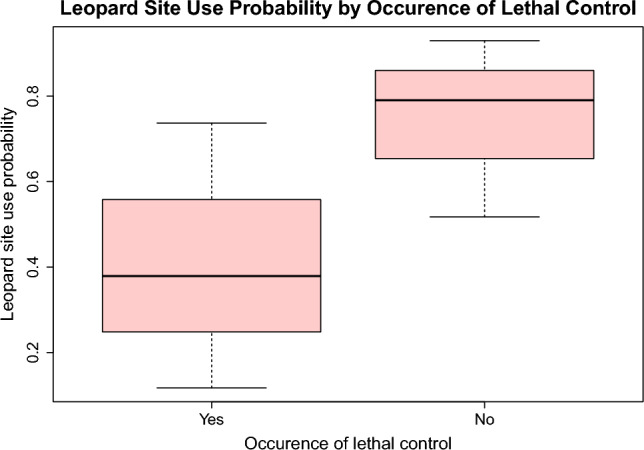
Leopard site use was considerably lower on properties that had reported to use lethal control compared to those that had not.

The best performing model included the effect of trail type on probability of detection and that of lethal control on probability of site use. We found a significant negative relationship between leopard site use probability and the use of lethal control on a property. Properties that reported not to use lethal control showed a considerably higher probability of site use (0.79 ± SE 0.11; 95% CI 0.52–0.93, *β* = 1.33), than those that have (0.38 ± SE 0.18; 95% CI 0.12–0.74, *β* = − 1.82) (Fig. 1 and Table 2, all model results in SI, Table S2). The goodness-of-fit test on the global model indicated there was no overdispersion (ĉ = 0.99) or lack of fit (*p* = 0.52).

**Table 2:** Model selection results for models with $\Delta {\varvec{A}}{\varvec{I}}{\varvec{C}}{\varvec{c}}<6$, excluding expanded versions of a better performing model.

Model Name	K	AICc	Delta AICc	AICcWt	LL
*Ρ* ~ (Trail type) *Ψ* ~ (lethal control)	5	380.80	0.00	0.25	− 184.1
*Ρ* ~ (Trail type) *Ψ* ~ (.)	4	381.25	0.45	0.20	− 185.79
Null	2	386.09	5.29	0.02	− 190.81

P describes the covariate modelled on probability of detection, Ψ describes the covariate modelled on habitat use with (.) indicating constant, K is the number of model parameters, AICc is Akaike’s Information Criterion weighted for small sample size, Delta AICc is the difference in AICc score between the best model and the model being compared, AICcWt is the AICc weight which describes each model’s proportion of total predictive power provided by the complete set of models assessed and LL is Log Likelihood which describes how likely the model is given the data.

Our results did not support significant relationships between probability of leopard site use with distance to buildings, kraals, roads, rivers and crop fields, nor prey availability, NDVI or human population density (all model results in SI, Table S2).

**Figure 2:**
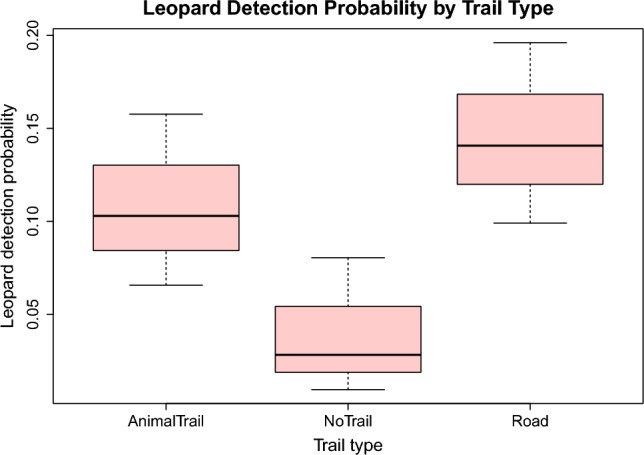
Detection probability differed across trail type, as expected, with roads and animal trails yielding greater detection probabilities than cameras not on trails.

Detection probability was lowest with no trails (*β* = − 1.19) but higher in areas with roads (*β* = 0.39) than in those with animal trails (*β* = -2.19) only (Fig. 2).

## Discussion

Our results suggest that leopards can and do coexist with humans in commercial farming environments in South Africa and adapt their habitat use to areas where risk of direct human persecution is lower. Our density estimate of 2.21 leopards/100km^2^ is similar to others in unprotected areas and farming landscapes^30–32^. Leopards appear to adapt their space use more to human pressure than prey availability, suggesting that they adjust their behaviour to survive in these anthropogenic landscapes. We begin by discussing our density estimate in the context of landowner opinion, before considering our results for leopard space use and the conservation implications of our findings.

While our population density was low in global terms, it is in line with other studies of leopards in similar fragmented landscapes in South Africa^32^ and even in some protected areas of Limpopo Province^30^. Faure et al.^31^ estimated 2.2 leopards/100km^2^ from the neighbouring Platjan farming area. Nonetheless, this contrasts with landowners who expected leopard density to be high^27^. Leopards are reportedly highly persecuted legally and illegally in the Alldays area^33^. It has been estimated that reproductive females make up 35% of retaliatory actions by landowners that result in leopard mortality^34^, resulting in reduced leopard survival and population viability outside protected areas^35^. While our female to male density ratio is not as high as observed elsewhere^36^, it suggests that the population should be viable, notwithstanding other influences, such as genetic diversity^35^. The observation of three cubs from two different females confirms that breeding is occurring, while a male cub identified from previous camera trapping (unpublished data) was observed as an adult, showing that some cubs are persisting to adulthood.

Agricultural intensification to meet the increasing needs of a growing human population is a key contributor to habitat fragmentation and biodiversity declines^37,38^. The Alldays area, as with many parts of Limpopo Province, has undergone widespread land-use changes in the last 40 years. Livestock farming, often considered harmful to natural habitat and prey species^39,40^, dominated the landscape for many years, and can lead to persecution of carnivores when competing with humans^28^. The emergence of game farming as a plausible economic industry and its subsequent growth has led to it becoming the dominant agricultural practice in the area, indirectly helping to promote elements of biodiversity conservation^41^. Not only have previously rare or extirpated ungulates returned to these areas in great numbers, including important preferred prey species, natural habitat to sustain these animals has also returned, providing the cover that many carnivores rely upon for successful hunting^42^ and sanctuary. Our habitat use results showed no direct relationship with vegetation cover, likely linked to its relative uniformity across the study area. Large carnivores, like leopards, have benefitted from the improved habitat quality that has resulted over the past four decades ^8^. Although game farming benefits natural habitat, it also fosters considerable intolerance towards large carnivores like leopards^15^. However, farmers in Alldays suggest that the land conversion away from livestock farming may have also resulted in greater tolerance of large carnivores ^33^. Historically, livestock farmers invested heavily in predator control. However, farmers believe that today’s game or mixed farming and “hobby” farming, which often have a lesser presence on their properties, have resulted in less investment in lethal control. This reduced pressure on leopard populations, enabling their numbers to increase^33^. These gaps in lethal control enable leopards to persist in the area despite the potential “attractive sink” effect of the environment.

Large carnivores recognising higher risk areas is not new^10,16–18^, but few studies have sought to understand large carnivore responses to human activity where the carnivores are not able to utilise a formally protected area as sanctuary^11^. Our space-use models found that reported lethal control on properties was the only factor measured influencing leopard space use. Space use dropped to 0.38 for properties that had reported to use lethal control, but was 0.79 for properties that had not. This suggests a spatial risk-avoidance strategy in areas with greater human pressure, similar to other carnivore populations^13,16,43^. Even where direct human pressure might be comparatively lower, large carnivores have demonstrated spatial risk-avoidance strategies, as with wolves in Europe ^16^. In other parts of Europe, it has also been suggested that where large carnivores are observed to coexist with human populations, it is as a result of human exodus from rural areas and the associated abandonment of agricultural land^44^. Farmers in Alldays anticipated similar behaviour. Considering leopards to be shy and skittish, farmers suggested that leopards used parts of their properties that minimised human contact, as seen elsewhere^45^—notably elevated areas such as koppies (rocky outcrops)—and that any human interactions with carnivores were most common at night^33^. Our study did not evaluate these topographical features, or activity patterns specifically. Nonetheless, the effect of lethal control by humans perhaps supersedes leopard responses to any other anthropogenic features that we did analyse (e.g. roads), and ultimately produced a similar pattern to that expected by farmers—the avoidance of areas with highest perceived risk. General game hunting for personal, commercial or culling reasons occurred on all bar one of the properties hosting camera trap stations. As such, it is not the wider act of hunting that was contributing to leopard adapting their space use; rather, habitat use seems to be affected by something more specific to the mortality risks for leopards.

No other covariates included in the study showed a significant impact on leopard space use. The absence of a relationship between space use and prey abundance has been reported in other studies^46,47^ and our results support Balme et al.’s^42^ suggestion that prey abundance is not always the most important factor in leopard population dynamics. Nevertheless, we chose only five prey species to represent prey species availability, all with a mass > 18kg, based on Hayward et al.^20^. Leopard are known to be very adaptable in their diet^20^ and a wider selection of prey species might have yielded different results^48^. While previous studies have suggested that human settlements influence leopard behaviour^46,49,50^, our camera trap stations were located at least 2.7km away from any settlement, likely minimising any potential influence. Instead, we considered the distance of camera traps from individual buildings as a covariate to account for micro-level human settlement effects. It might be the case that leopards continue to use these spaces but adapt temporally to avoid greater human presence as observed in Indian urban spaces^18^. This might also be applicable to kraals (enclosures or pens, typically used to confine livestock for husbandry or protection purposes) and crop fields, where presence of humans is likely to primarily influence leopard behaviour during the day when herders, farmers, and pickers are present, as seen with lions^51^. Moreover, livestock numbers in the study area were relatively low, and the use of enclosures might only have temporary or minimal effects on leopard activity; other camera traps at kraals recorded only sporadic nocturnal visits by leopards (JM and CL, unpublished data). Farmers also employ additional protective strategies for their livestock, game and crops, including specific placement of kraals near farm buildings, livestock guardian animals and varying levels of fencing. Fencing is particularly prevalent, but we observed individual leopards on multiple properties, suggesting fencing does not completely constrain their movement. However, some fencing types, such as high voltage electric fences, might still deter leopards and encourage them to adapt their space-use, forcing them, for instance, to use roads or travel closer to buildings that they might otherwise have avoided.

The mixed land use also brings differing attitudes and approaches to large carnivore control. As noted in other locations of human-carnivore coexistence, within these landscapes there is still a requirement that various local, cultural, and regulatory practices are upheld to sustain it^18,52,53^. Maintaining and encouraging greater use of traditional livestock protection measures, such as livestock guardian dogs and kraaling animals at night, are important non-lethal tools to minimise large carnivore depredation on livestock^27,53,54^. The latter method, especially coupled with electric fencing of kraals, can also be employed for protecting exotic breeding game. This helps to foster a greater number of landowners and properties that can become perceived safer spaces for leopards to traverse.

The density and space use pattern of leopards in our study emphasises their remarkable ability to coexist in close proximity to humans and their resilience to direct human pressure, particularly attempts to exterminate their presence from the area^33^. To ensure successful conservation of large carnivores, it is crucial to prioritise connected landscapes^45^. Our results suggest a mixed mosaic landscape and wider adoption of management approaches that minimise lethal control could provide this connectivity by providing smaller properties as safe stepping stones or ‘wild spaces’, without requirement for larger-scale protected areas and management outlays^55–58^. This underscores the potential for these regions to serve as vital corridors linking large carnivore populations. Allowing extensive areas to continue to be used for agriculture^59^, while also providing places of sanctuary and greater connectivity allows leopards and large carnivores to persist in and navigate these transformed landscapes. This balances national and global food supply requirements with biodiversity needs.

## Methodology

### Study site

**Figure 3:**
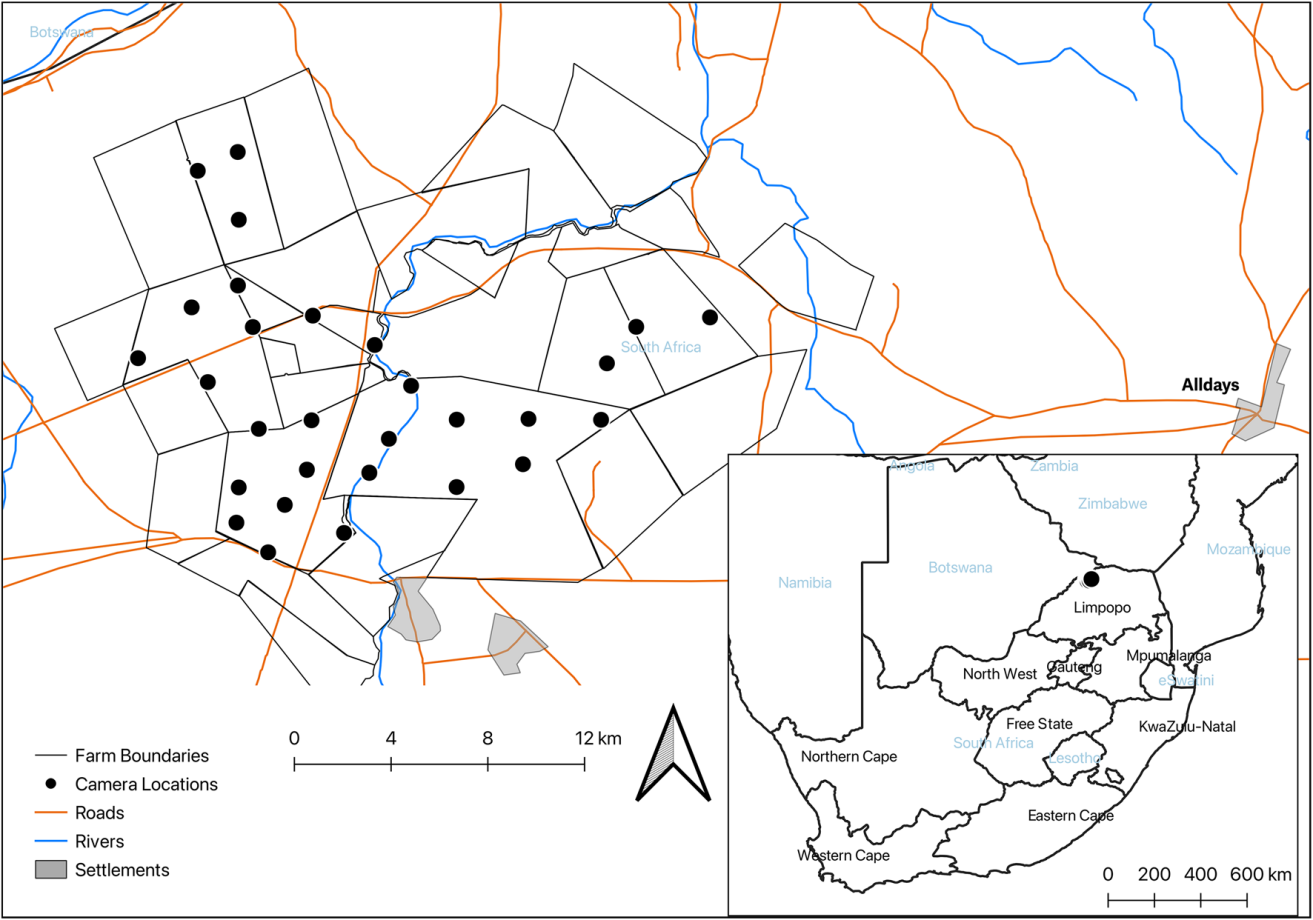
Location of the study area and the camera survey on farmland in the Blouberg Municipality of Limpopo province, South Africa.

The study took place near Alldays in the Blouberg Municipality of Limpopo Province, South Africa (Fig. 3; central coordinates: -22.674960, 29.020938). The survey area included 21 properties, totalling c.150km^2^, with camera traps placed on nine of these (Fig. 3). The wider area around Alldays is mixed land-use, dominated by game farming (for breeding, sales and hunting), crop farming and livestock farming, with some eco-tourism reserves. Of the nine properties on which our cameras were placed, three were mixed land use (crops, game and livestock), two were used for game and livestock, one was used for game and crop farming, two had livestock only and one had game only. All properties were fenced, predominantly with electrified game fence (~ 2.4m high), but some also used lower livestock fencing. Despite these barriers, fence lines were regularly crossed, including by leopards, using holes in or under the fences created by other species, particularly warthogs (*Phacochoerus africanus*).

The Mogalakwena River dissects the study site and provided many properties with a natural water source, while also providing wildlife with a safer route underneath the main road used by regular agricultural and large mining vehicle traffic^60^. The area has a semi-arid climate^61^ experiencing dry winters (April-September), with most of the mean annual 650mm rainfall falling in the summer months (October–March). The average daily minimum temperature is 13 Celsius in June and July, with the average temperature high of 33 Celsius in November^61^. The vegetation in the Alldays area is classified as Limpopo Sweet Bushveld^62^.

Predator control occurs in the region, using both legal and illegal methods^33^; illegal methods, in particular, are often non-selective (e.g., snaring and trapping), affecting a wide range of species^63^. Legal methods include trophy hunting and removal of problem animals through damage causing animal (DCA) permits^34^. Landowners also use a number of mitigation strategies, including kraals, electrified kraals, herders and livestock guardian dogs to deter carnivore species from specific areas of their property. These control methods influence the local environment for all animals in the area, and reflect the province more widely^29,64^.

### Camera trap setup

A camera trap survey was conducted from 4th June to 2nd September 2020 to estimate leopard population density using Spatially Explicit Capture-Recapture (SECR) and site-use using occupancy modelling. We created a 2km grid over the survey area and deployed 58 camera traps at 29 stations, with one station per grid square. Stations were an average of 2.04 km apart (range 1.5–2.7 km) (Fig. 3). This spacing was based on the average home range size of female leopards, minimising the chance that an entire leopard home range falls between camera placements (probability of detection ($\rho )>0$)^65,66^. Due to property access not being granted by some landowners, we were unable to extend the survey more widely. Nonetheless, we were able to survey across the main property land use types in the area, as well as across varying attitudes to carnivore conflict mitigation strategies.

Locations of camera stations were chosen to maximise probability of detecting leopards, with selection of sites guided by successful capture locations in past camera trapping, and placements along roads, trails and dry river beds that showed evidence of leopard presence through tracks and scat, reinforced by local farmer observations. Cameras were placed on trees opposite each other in pairs 5-10m apart, at a height of c. 40cm, and level with the ground to maximise chance of detection. Every camera station included at least one Browning Strike Force HD Pro Model BTC-5HDP for consistency across the study site. The pair was then made up of either another Browning or a Bushnell Trophy Cam HD. Settings between the camera models were set to match as closely as possible, with trigger time between consecutive photographic events set to the minimum possible and all cameras set to take three consecutive images for every trigger event, to improve the likelihood of capturing clear images of both flanks and the possibility of achieving individual identification from coat patterns. Cameras were visited at least monthly, in line with local COVID-19 restrictions in place at the time, to change memory cards and batteries, if necessary, and to check cameras were functioning properly with no vegetation obstructing their field of view. The survey period was limited to 90 days to ensure the study did not violate assumptions of closure^67^.

### Covariate data

Trail types have been shown to influence carnivore detection probability^68^ and we defined trail types as animal trails (AT), no trail (NT) and farm road (RD) for analysis.

We considered 10 anthropogenic and environmental covariates that could influence leopard habitat selection in the area. Additional detail on the calculation, definition, and rationale for inclusion of covariates can be found in Table 3. Distances to public roads, rivers, crop fields, kraals and buildings were calculated by using line and polygon layers created in *QGIS 3.22.9*^69^ from GPS data collected by the Primate and Predator Project over several years and supplemented with data from freely available satellite images from AGIS (1: 50 000) and Google (15–30m per pixel). Distances from these features to the camera trap stations were measured using the v.distance tool from the *GRASS 8.2* plugin^70^. Human population density data for 2020 was taken from the Socioeconomic Data and Applications Centre^71^, mapped at 1km grid square granularity. We used the mean of the 16-day NDVI composites from 1st of June 2020 to 1st October 2020 at 250m pixel size (MODIS MOD13Q1)^72^, extracted from Google Earth Engine^73^.

**Table 3:** Description﻿s of covariates used on probability of detection in our Spatially Explicit Capture-Recapture analysis (g0) and probability of detection in our habitat use analysis (P), and covariates used on habitat use (Ψ), with field name, data source and justification for including them in analysis. CT =  camera trap.

Covariate	Description	Name	Modelled effect on	Data source	Justification
Sex	Sex of individual leopards identified in study area	*Sex*	*g0*	Camera Trap	Male leopard typically have a larger home range than females^75^ and have been shown to utilise different habitat types ^35,76^
Trail type	Trail type defined as: Animal trails (AT), No trail (NT) and Farm road (RD)	*Trail_Type*	*g0, p*	Camera Trap	Detection probabilities can differ when camera traps are placed on roads, game trails or no trails^48,68^, although see Abade et al.^46^
Lethal control	Occurrence of lethal control was determined for each CT using a binary variable	*Control*	*Ψ*	Lucas^33^	Populations have been shown to be demographically sensitive to over-harvesting from trophy hunting ^23^
Livestock presence	Livestock presence was determined for each CT as a binary variable based on whether livestock were captured on that CT during the survey	*Livestock_YN*	*Ψ*	Camera Trap	Livestock presence negatively influences leopard space use ^46^, potentially as an avoidance strategy of herders and intensive human activity or the subsequent overgrazing and prey displacement from livestock presence^46^. Leopards have also been noted to hunt livestock^28^, which may attract them to livestock presence
Prey availability	Prey availability was determined for each CT as mean catch per-unit effort (CPUE) ^48^ for five key species: bushbuck (*Tragelaphus scriptus*), common duiker (*Sylvicapra grimmia*), greater kudu (*Tragelaphus strepsiceros*), impala (*Aepyceros melampus*) and warthog (*Phacochoerus africanus*)	*CPUE_mean.Total*	*Ψ*	Camera Trap	Prey availability positively influences leopard space use^11,48,49,77^ and also density^78,79^, although this is not always the case^46,47^. Prey availability can also reflect the indirect effect of prey depletion by human exploitation^80^
Distance to roads	Distances from public roads to the CT stations measured in QGIS using the v.distance tool	*Distance_to_Roads*	*Ψ*	GIS–AGIS dataset 1: 50 000 (Downloaded from: https://hub.arcgis.com/datasets/NRF-SAEON::south-african-roads/about)	Human disturbance has been negatively associated with leopard habitat use, with roads having the potential to cause direct and indirect impacts on leopards^35^. Direct traffic collisions have been found in road mortality studies^35^
Distance to river	Distances from rivers to the CT stations measured in QGIS using the v.distance tool	*Distance_to_Rivers*	*Ψ*	GIS–AGIS dataset 1: 50 000 (Downloaded from: https://hub.arcgis.com/datasets/865fb8ed525045ee862169b1e031299a_0/about)	While distance to water generally has no significant influence on leopard abundance or habitat use^50,81^, there is evidence for leopards preferring habitat nearer rivers^82^ and riverine habitat^48^, which may also increase the likelihood of leopard predation^28^
Distance to kraals	Distances from kraals to the CT stations measured in QGIS using the v.distance tool	*Distance_to_Kraals*	*Ψ*	GIS–Data collected by the Primate and Predator Project	Studies have demonstrated that livestock presence negatively influences leopard space use^46^, while landowner perception of real or perceived threats to livestock^28^ suggests leopards may not avoid these areas
Distance to crop fields	Distances from crop fields to the CT stations measured in QGIS using the v.distance tool	*Distance_to_Cropfields*	*Ψ*	GIS-Data collected by the Primate and Predator Project and supplemented with data from Google satellite imagery (15–30 m per pixel)	Crop fields have been shown to increase leopard space use^83^, due to increased availability of smaller prey species^84^ in crop farming areas, but human presence on fields may deter daytime use
Distance to buildings	Distances from buildings to the CT stations measured in QGIS using the v.distance tool	*Distance_to_Buildings*	*Ψ*	GIS-Data collected by the Primate and Predator Project and supplemented with data from Google satellite imagery (15–30 m per pixel)	Human settlement and activity have been shown as one of the most significant negative predictors of leopard space use, but urban studies in India suggests it can also be positive^46,49,50,85,86^
NDVI	Normalised Difference Vegetation Index to quantify vegetation greenness and vegetation density at a 250m pixel basis based on mean of 16-day composites between 01/06/2020 and 01/10/2020	*NDVI*	*Ψ*	MODIS/TERRA MOD13Q1 Vegetation Indices from Google Earth Engine^72^	Vegetation differences have been shown to influence leopard habitat use^48^, with use increasing with greater vegetation density for hunting benefits^42^
Human population density	The population data is produced as a global raster using 30 arc-second granularity (approximately 1km)	*Human_Population_Density*	*Ψ*	The Gridded World Population (GPWv4) data for 2020 taken from the Socioeconomic Data and Applications Centre (SEDAC)^71^	Human disturbance has often been linked to reduced leopard habitat use and density, although not always^24^. There has also been an expectation that higher human population density results in greater levels of illegal bushmeat poaching and snaring that will negatively influence leopard habitat use^35^

Data were available for the number of livestock on each property from farmers; since many of these remained in kraals or specific areas of the property, we used camera trap data to determine livestock presence in a particular location during the study, using a binary variable indicating presence (1) or absence (0). Prey availability was determined from the camera trap data by calculating a catch per-unit effort (CPUE) index for each camera location. CPUE was calculated as in Burton et al.^48^, multiplying the number of independent captures of each prey species at the individual camera stations by their average mass, based on standard guide data from Tacutu et al.^74^, divided by the camera trap sampling effort and standardised for 100 days of camera trapping effort. Independence was determined as five minute intervals as in Burton et al.^48^. Based on Hayward et al.^20^, five species were used to represent the availability of preferred prey species: bushbuck (*Tragelaphus scriptus*), common duiker (*Sylvicapra grimmia*), greater kudu (*Tragelaphus strepsiceros*), impala (*Aepyceros melampus*) and warthog. The mean prey availability was then calculated from the combined prey CPUEs for each camera location.

Finally, we used farmer responses from the interview data (n = 20) from Lucas^33^ to determine whether any form of lethal control had occurred on a property in the past 10 years. Interviews were semi-structured, with participants recruited through trusted collaborators and by attending local events such as game and livestock auctions (full details in^33^). Interviewees were not specifically asked about lethal control measures but was voluntarily mentioned by participants. Where use of lethal control methods were mentioned, the exact details of the lethal control used (i.e., using hunting permits, DCA permits or any other means of control) were not requested. Data collection protocols for the interviews was reviewed and approved by Nottingham Trent University School of Animal, Rural and Environmental Sciences Ethical Review Group (ARE880) and all interviews were performed in accordance with the relevant guidelines and regulations of Data Protection laws in the UK and South Africa, in adherence with this approval. The participants provided their written informed consent to participate in this study and were aware of their right to withdraw.

### Leopard density analysis

Individual leopards were identified based on their unique coat patterns, using manual verification by two researchers (JM and CL). Only ID’s where both researchers agreed were included in capture histories. As recommended for predominantly nocturnal species, sampling occasions were defined as the 24-h periods between consecutive middays. This avoids the ‘midnight problem’ of recording an animal as two separate captures when it is photographed on both sides of midnight at the same location. Spatial capture histories for all identified individuals were constructed for all 90 sampling occasions. Leopard density was estimated using maximum likelihood to fit SECR models with the package *secr 4.5.8*^87^ in *R 4.2.2*^88^.

In SECR, density is estimated across a habitat mask, which comprises the explicit spatial extent where sampling occurs, including a buffer area around the outer camera traps, encompassing the entire surveyed area. This inclusion acknowledges that individuals captured in the survey may originate from beyond the perimeter of these camera traps. The buffer zone is essential to accommodate the extra area from which leopards may be captured^65,89^.

To ensure the buffer size was sufficient to include all activity centres of individuals available for capture by the cameras, we followed Faure et al.’s^31^ approach, testing buffer size in 2.5km increments, ultimately assuming buffer size was large enough when density estimates plateaued with increasing size and when it was a minimum of three times greater than the spatial scale parameter ($\sigma$) (12.5km). The SECR likelihood is assessed by summing values at points on the habitat mask, with each point representing a grid cell of potential home range centres or occupied habitat. We used a home range centre spacing of 100m for all models and assumed that leopards were able to utilise all anthropogenically modified areas in the study area (i.e., including crop fields); as such, all home range centres were considered suitable habitat in the state-space input file.

We fitted the data with half-normal, hazard rate and negative exponential detection functions, retaining the function with the lowest Akaike information criterion, corrected for small sample size (AICc). The negative exponential was the best supported detection function and this was used in all subsequent models. We modelled the relationships between trail type and detection probability (g0), and sex and g0, and also fitted two learned-response models, where probability of detection is influenced by previous captures across the survey (*b*) and where probability of detection is influenced by specific detectors (*bk*). We used the derived function in secr to obtain g0 and density estimates, and the MMDM function in *secr* to estimate the mean maximum distance moved.

### Leopard site use analysis

It is likely that designing the survey to prevent an individual leopard’s entire home range falling between cameras precludes detections being independent, as the same animal could be captured at multiple locations. This relaxes the assumption of strict closure meaning we interpreted the results of occupancy probability as site use probability^90^, which was the original focus.

To estimate site use, we used a single season occupancy modelling approach to fit occupancy models using the package *unmarked*^91^ in R. We relaxed the temporal extent of the sampling, aggregating to 30 three-day sampling periods to reduce temporal autocorrelation^92^.

All continuous variables were scaled to between 0 and 1. We included trail type as a covariate on space use detection probability *P*, while all other covariates were assumed to bear only on occupancy (Ψ). AICc was used to rank competing models with different covariate sets. The sign of the beta coefficient estimates of the covariates represented the direction of the covariate impact. We considered all models that had a $\Delta AICc<6$, excluding those that were expanded versions of a better performing model (i.e., where adding parameters led to a larger AICc), as models with some empirical support^93,94^. The best fitting model was used to predict leopard site use. Model fit and overdispersion were evaluated using the sum of squared residuals and 10,000 parametric bootstraps.

### Supplementary Information

Supplementary Tables.

## Data Availability

The data and code used in this article will be openly available on OSF https://osf.io/uwfvj/?view_only=e50f1c0bd09e433194ba2055bd04faae.
