# Supplementary material for: Leopard density and determinants of space use in a farming landscape in South Africa
Source: Sci Rep. 2024 May 8;14:10562. doi: 10.1038/s41598-024-61013-6 (PMC11079070; doi:10.1038/s41598-024-61013-6)
Supplement: Supplementary file 1 — Supplementary Tables. [file 41598_2024_61013_MOESM1_ESM.pdf]

Leopard density and determinants of space use in a farming landscape in South Africa

Authors

McKaughan, J.E.T.[1,2,3*], Stephens, P.A.[2], Lucas, C.[4], Guichard-Kruger, N.[3], Guichard-Kruger, F.[3], Hill, R.A.[1,3,5]

[1]Department of Anthropology, Durham University, South Road, Durham, DH1 3LE, United Kingdom

[2]Conservation Ecology Group, Department of Biosciences, Durham University, South Road, Durham, DH1 3LE, United Kingdom

[3]Primate and Predator Project, Alldays Wildlife and Communities Research Centre, Campfornis Game Farm, Alldays, South Africa

[4]School of Animal, Rural and Environmental Sciences, Nottingham Trent University, Southwell, United Kingdom

[5]Department of Biological Sciences, Faculty of Science, Engineering and Agriculture, University of Venda, Private Bag X5050, Thohoyandou 0950, South Africa.

Table S1. Model selection results for all models of our secr analysis with their respective density estimates. g0 describes the covariate modelled on probability of detection, sigma remained constant (~1), npar is the number of model parameters, logLik is Log Likelihood which describes how likely the model is given the data, AICc is Akaike's Information Criterion weighted for small sample size, dAICc is the delta AICc which is the difference in AICc score between the best model and the model being compared, AICcwt is the AICc weight which describes each model's proportion of total predictive power provided by the complete set of models assessed, SE is the standard error.

**Model**	**npar**	**logLik**	**AICc**	**dAICc**	**AICcwt**	**Density ± SE (leopards/100km^2)**
g0~bk sigma~1	3	-308.5061	625.679	0.000	0.7506	2.21 ± 0.74
g0~1 sigma~1 (NULL)	2	-311.9440	629.088	3.409	0.1365	2.21 ± 0.72
g0~b sigma~1	3	-311.3044	631.275	5.596	0.0457	3.01 ± 1.48
g0~Trail_Type sigma~1	3	-311.3637	631.394	5.715	0.0431	2.26 ± 0.73
g0~Sex sigma~1	3	-311.9440	632.555	6.876	0.0241	2.21 ± 0.72
g0~Trail_Type + Sex sigma~1	4	-311.3637	635.727	10.048	0.000	2.26 ± 0.74

Table S2. Model selection results for models with $\Delta AICc < 6$, including expanded versions of a better performing model. P describes the covariate modelled on probability of detection, Ψ describes the covariate modelled on habitat use with (.) indicating constant, K is the number of model parameters, AICc is Akaike's Information Criterion weighted for small sample size, AICcWt is the AICc weight which describes each model's proportion of total predictive power provided by the complete set of models assessed, Cum.Wt is the sum of the AICc weights and LL is Log Likelihood which describes how likely the model is given the data. The β-coefficient quantifies the effect of the covariate on the likelihood of a species using a particular space, SE± is the standard error, with LCI and UCI the 95% lower and upper confidence intervals respectively.

**Model Name**	**K**	**AICc**	**Delta AICc**	**AICcWt**	**Cum.Wt**	**LL**
P ~ (Trail Type) Ψ ~ (Lethal Control)	5	380.80	0.00	0.25	0.25	-184.1
P ~ (Trail Type) Ψ ~ (.)	4	381.25	0.45	0.20	0.46	-185.79
P ~ (Trail Type) Ψ ~ (Distance to Buildings)	5	382.48	1.68	0.11	0.57	-184.94
P ~ (Trail Type) Ψ ~ (Vegetation Cover)	5	383.70	2.90	0.06	0.62	-185.55
P ~ (Trail Type) Ψ ~ (Total Prey)	5	383.76	2.95	0.06	0.68	-185.57
P ~ (Trail Type) Ψ ~ (Distance to Crops)	5	383.82	3.02	0.06	0.74	-185.61
P ~ (Trail Type) Ψ ~ (Distance to Rivers)	5	383.98	3.18	0.05	0.79	-185.69
P ~ (Trail Type) Ψ ~ (Distance to Kraals)	5	384.10	3.30	0.05	0.84	-185.75
P ~ (Trail Type) Ψ ~ (Human Population Density)	5	384.10	3.30	0.05	0.89	-185.75
P ~ (Trail Type) Ψ ~ (Distance to Roads)	5	384.14	3.34	0.05	0.94	-185.77
P ~ (Trail Type) Ψ ~ (Livestock Presence)	5	384.18	3.38	0.05	0.98	-185.78
Null	2	386.09	5.29	0.02	1.00	-190.81

**Parameters**	**β-coefficient**	**SE±**	**LCI**	**UCI**
Lethal control	-1.82	1.0	-3.46	-0.18
No lethal control	1.33	0.64	0.27	2.38
Distance to buildings	-7.44	5.83	-17.04	2.16
Vegetation cover	10.9	17.58	-18.03	39.79
Total prey	0.45	0.79	-0.84	1.74
Distance to crops	-1.80	2.95	-6.65	3.05
Distance to rivers	-0.87	1.90	-4.0	2.25
Distance to kraals	0.93	3.03	-4.05	5.91
Human population density	-1.48	4.78	-9.34	6.39
Distance to roads	1.36	5.96	-8.43	11.16
Livestock presence	0.14	1.02	-1.54	1.83
